# Growth hormone therapy and near-final height in *SHOX* deficiency and turner syndrome: a real-world single-center retrospective cohort study

**DOI:** 10.1007/s00431-026-07135-7

**Published:** 2026-06-05

**Authors:** Mehmet Ali Oktay, Esra Döğer, Aylin Kılınç Uğurlu, Gülsüm Kayhan, Meral Yirmibeş Karaoğuz, Mahmut Orhun Çamurdan, Aysun Bideci

**Affiliations:** 1 Faculty of Medicine Department of Pediatric Endocrinology, Gazi University, Ankara, Turkey; 2 Faculty of Medicine Department of Genetics, Gazi University, Ankara, Turkey

**Keywords:** *SHOX* deficiency, Turner syndrome, Near final height, Growth hormone therapy

## Abstract

Short stature homeobox‐containing (*SHOX)*-gene haploinsufficiency causes short stature both in isolated *SHOX* deficiency and in Turner syndrome (TS), yet head-to-head data on growth-hormone (GH) outcomes remain scarce. We therefore compared growth response and near-final height (NFH) after GH therapy between girls with *SHOX* deficiency and those with TS. Single-centre retrospective cohort study. In this single-centre retrospective cohort, we reviewed 44 pre-pubertal girls who received daily GH (mean 0.045 mg/kg/day) between 2015 and 2024 and subsequently reached NFH (bone age ≥ 14 y; height velocity < 2 cm/year). Ten had molecularly confirmed *SHOX* deficiency and 34 had karyotype-proven TS. Baseline auxology, annual height velocity, pubertal timing, treatment duration, NFH standard-deviation score (SDS) and total height SDS gain were analysed. Mean chronological and bone ages at GH initiation were similar (10.7 ± 1.9 vs 10.4** ± **2.7 years). Baseline height SDS was lower in TS (–3.51 ± 1.0) than in *SHOX* (**–**2.66 ± 0.8). First-year growth velocity was higher in *SHOX* (8.55 ± 1.4 cm/year) than TS (6.72 ± 1.9 cm/year; *p* = 0.01). Puberty began earlier in *SHOX* (11.6 ± 1.3 years) than TS (13.7 ± 1.5 years; *p* < 0.01), and GH exposure was shorter (2.00 ± 0.4 vs 4.38 ± 2.3; *p* < 0.01). Observed NFH-SDS was similar between groups (*SHOX* − 2.46 ± 1.2 vs TS − 2.47 ± 1.0). Unadjusted height-SDS gain was greater in TS (1.04 ± 0.1 vs 0.20 ± 0.2**;** *p* = 0.04). In exploratory ANCOVA models adjusting for baseline age, baseline height SDS, and GH treatment duration**,** adjusted height-SDS gain did not differ significantly between groups.

*Conclusions*: TS was associated with a greater unadjusted height-SDS gain, whereas *SHOX* deficiency was characterized by earlier puberty and shorter GH exposure. Although observed NFH-SDS was similar, these findings highlight how differences in pubertal timing and treatment duration may influence growth outcomes in routine clinical practice and should be interpreted as reflecting real-world growth trajectories rather than intrinsic biological responsiveness to GH therapy.

**Table:** 

**What is Known:** • *Growth hormone therapy is an established treatment for short stature in both SHOX deficiency and Turner syndrome.* • *Treatment response is influenced by several factors, including age at treatment initiation and pubertal development.*	
**What is New:** • *This study provides a real-world comparison of growth hormone treatment outcomes in girls with SHOX deficiency and Turner syndrome.* • *Spontaneous pubertal progression in SHOX* *deficiency versus medically induced puberty in Turner syndrome may substantially influence the duration of the therapeutic growth window and the observed growth response.*

**What is Known:**

• *Growth hormone therapy is an established treatment for short stature in both SHOX deficiency and Turner syndrome.*

• *Treatment response is influenced by several factors, including age at treatment initiation and pubertal development.*

**What is New:**

• *This study provides a real-world comparison of growth hormone treatment outcomes in girls with SHOX deficiency and Turner syndrome.*

• *Spontaneous pubertal progression in SHOX* *deficiency versus medically induced puberty in Turner syndrome may substantially influence the duration of the therapeutic growth window and the observed growth response.*

##  Significance statement

This study directly compares growth hormone therapy outcomes between two distinct genetic causes of short stature: short stature homeobox‐containing (*SHOX)* deficiency and Turner syndrome (TS). Although *SHOX* haploinsufficiency underlies short stature in both conditions, data comparing treatment response head-to-head are limited. Our findings show that observed near-final height standard deviation score (NFH-SDS) was similar between groups despite marked differences in growth trajectories, pubertal timing, and treatment duration**.** These results emphasize that differences in pubertal timing substantially influence the duration of the available growth window. By reflecting routine clinical practice rather than protocol-driven treatment conditions, this study provides real-world insight into how pubertal timing and treatment duration shape growth outcomes in genetic short stature.

## Introduction

Short stature is one of the most frequent reasons for endocrine evaluation in childhood, and genetic factors contribute substantially to its etiology. Among these, the *SHOX* gene located in the pseudoautosomal region 1 (PAR1) at Xp22.3/Yp11.3 on the distal tips of the X and Y chromosomes escapes X-inactivation and plays a pivotal role in regulating linear growth [1, 2]. *SHOX* supports longitudinal bone development primarily by controlling chondrocyte proliferation and differentiation within the growth plate [1, 3].

Haploinsufficiency of *SHOX* presents as a clinically heterogeneous spectrum. At its severe end lies Langer mesomelic dysplasia (LMD); intermediate severity is represented by Léri–Weill dyschondrosteosis (LWD); and milder forms may present as isolated short stature without overt skeletal abnormalities [1–4]. LWD is characterized by mesomelic shortening of the lower limbs, Madelung deformity, and various skeletal dysmorphisms [1, 2, 4, 5]. Inherited in a pseudoautosomal-dominant pattern, LWD often exhibits a more pronounced phenotype in females [5, 6].

*SHOX* haploinsufficiency is the principal driver of the short-stature phenotype in TS [1, 7]. TS most commonly affects individuals with a 45, X karyotype; partial or complete loss of the second X chromosome reduces *SHOX* expression in growth plates, leading to progressive short stature that begins in utero and is accentuated by the absence of a pubertal growth spurt [7–10].

Growth-hormone (GH) therapy is widely employed to treat short stature in both *SHOX* deficiency and TS [7, 11, 12]. Numerous studies have demonstrated significant improvements in growth velocity, height SDS, and adult or near-final height in both conditions [8, 10, 13, 14]. However, most studies have evaluated these disorders separately, and direct comparisons of growth outcomes between *SHOX* deficiency and TS remain limited, particularly in real-world clinical settings. Moreover, differences in pubertal timing and treatment duration between the two conditions may substantially influence the duration of the growth window and the observed response to GH therapy.

The present study therefore compares the effects of GH therapy on NFH in patients with *SHOX* deficiency and TS in a real-world clinical cohort**.** Rather than attempting to establish biological equivalence of intrinsic GH responsiveness, this study aims to explore how differences in pubertal progression and treatment duration shape observed growth outcomes in routine clinical practice.

## Methods

This retrospective analysis included 44 female patients, 10 with genetically confirmed *SHOX* deficiency and 34 with karyotype-proven TS, who had completed recombinant human GH therapy at a mean dose of 0.045 mg/kg/day and subsequently reached NFH bone age. NFH was defined as a bone age (BA) ≥ 14 years and an annual height velocity < 2 cm/year. GH therapy was discontinued when patients fulfilled these predefined NFH criteria based on skeletal maturity (BA ≥ 14 years) and growth deceleration (height velocity < 2 cm/year), rather than at a fixed chronological age (CA). Height SDS at NFH was calculated based on chronological age at the time of assessment rather than projected adult height at age 18 years.

Eligible patients had started GH treatment while still pre-pubertal (Tanner stage 1) and fulfilled one of two auxological criteria at baseline: (i) height below the 3rd percentile or (ii) height between the 3rd and 10th percentiles combined with a height velocity below the 25th percentile for age and sex. Pubertal onset was recorded when breast development reached Tanner stage 2 on clinical examination. In patients with TS, pubertal onset was frequently induced with estrogen replacement therapy; therefore, age at Tanner stage M2 reflects timing of estrogen initiation rather than spontaneous puberty. Pubertal induction was performed using oral estradiol valerate (Estrofem). Estrogen therapy was generally initiated at approximately 0.25 mg/day and gradually increased approximately every six months according to a standardized institutional protocol until a target full-replacement dose of 2 mg/day was achieved. Although the age at pubertal induction varied between patients, the same dose escalation schedule was applied throughout the TS cohort without major individual variations in dose escalation timing.

Two experienced pediatric endocrinologists assessed BA independently using the Greulich–Pyle atlas [15]. When discrepancies exceeded 0.5 years, BA was reassessed jointly until consensus was reached. Patients were excluded if they had coeliac disease, chronic systemic disorders affecting growth, additional genetic syndromes, prolonged systemic glucocorticoid therapy, or documented non-adherence to GH injections.

### Statistical analysis

Statistical analyses were performed using IBM SPSS Statistics, version 22.0 (IBM Corp., Armonk, NY, USA). Height SDS, body-mass index (BMI) SDS, and height gain SDS were calculated using national Turkish reference data [16]. Birth weight SDS and birth length SDS were also calculated using gestational age and sex-adjusted national reference standards [16]. Height velocity SDS was determined according to age and sex-specific reference standards derived from the same population data. Insulin-like growth factor-1 (IGF-1) SDS and insulin-like growth factor binding protein-3 (IGFBP-3) SDS were calculated using age- and sex-adjusted laboratory reference ranges provided by the assay manufacturer and validated for use in the pediatric population. The distribution of continuous variables was assessed using the Shapiro–Wilk test. All continuous variables presented in the tables were normally distributed and are therefore expressed as mean ± SD. Between-group comparisons were performed using independent-samples t tests; non-parametric tests (Mann–Whitney U) were reserved for variables deviating from normality, if applicable. Within-group comparisons were performed using paired t-tests for normally distributed variables and Wilcoxon signed-rank tests for non-normally distributed paired variables.

To account for differences in baseline auxological characteristics and treatment duration between groups, adjusted analyses using analysis of covariance (ANCOVA) were performed to minimize potential bias arising from non-homogeneous baseline features. Absolute height gain (cm) and height-SDS gain were further explored using ANCOVA, with diagnosis group entered as a fixed factor. Two ANCOVA models were used: (i) absolute height gain (cm) adjusted for baseline age, baseline height (cm), and GH treatment duration; and (ii) height-SDS gain adjusted for baseline age, baseline height SDS, and GH treatment duration. Covariates were selected a priori based on known determinants of GH response and to account for baseline auxological differences. Adjusted values reported in the Results correspond to estimated marginal means derived from the ANCOVA models rather than directly observed measurements. Prior to ANCOVA, model assumptions including linearity and homogeneity of regression slopes were evaluated and found to be acceptable. Given the small sample size of the *SHOX* deficiency cohort, results of multivariable models were interpreted cautiously and considered exploratory in nature. All statistical tests were two-sided, and a *p* value < 0.05 was considered statistically significant.

### Ethics approval and consent to participate

This study was approved by the Clinical Research Ethics Committee of Gazi University (Approval Date: January 28, 2025; Approval Number: 2025–165). The study was conducted in accordance with the ethical standards of the institutional research committee and the principles of the Declaration of Helsinki. Written informed consent was obtained from all participants and/or their legal guardians prior to inclusion in the study**.** Permission to conduct the study was obtained from the relevant institutional authorities prior to data collection.

## Results

### Baseline characteristics

All participants were female: 10 in the *SHOX* deficiency group and 34 in the TS group. Within the *SHOX* cohort, seven patients (70%) had LWD, whereas three presented with short stature without classical skeletal features of LWD*.* Madelung deformity was clinically evident in 7/10 SHOX patients (all LWD cases) and in 0/34 TS patients. Molecular testing identified deletions involving *SHOX* or its regulatory regions in seven patients, two nonsense variants, and one missense variant; all variants were classified as pathogenic/likely pathogenic according to American College of Medical Genetics and Genomics (ACMG) criteria.

Among the 34 TS patients, 17 (50.0%) had classic 45, X monosomy. Thirteen (38.2%) exhibited various mosaic karyotypes. Two patients (5.9%) harbored an isochromosome Xq [i(Xq)], and two (5.9%) had structural abnormalities such as del(X) or ishder(X). Ring-X [r(X)] chromosomes occurred only within mosaic karyotypes and were not classified as a separate subgroup.

Age at GH initiation did not differ between groups (10.7 ± 1.9 y in *SHOX* vs 10.4 ± 2.7 y in TS; *p* = 0.96). Baseline height SDS averaged − 2.66 ± 0.8 in *SHOX* patients and − 3.51 ± 1.0 in TS patients (*p* = 0.08). Birth weight SDS, birth length SDS, chronological age, BA, initial height (cm), BMI, IGF-1 SDS, IGFBP-3 SDS, and pre-treatment height velocity showed no significant inter-group differences (*p* > 0.05). None of the patients in either group were born small for gestational age (SGA) (Table 1).

**Table 1:** Baseline characteristics at initiation of growth hormone therapy in patients with *SHOX* deficiency and Turner syndrome

Variable	SHOX	Turner	P
Birth weight (SDS)	− 0.2 ± 1.2	− 0.3 ± 0.9	0.60
Birth length (SDS)	− 0.75 ± 0.9	− 0.75 ± 1.0	0.90
Chronological age (years)	10.7 ± 1.9	10.4 ± 2.7	0.96
Bone age (years)	10.6 ± 0.7	9.05 ± 2.6	0.35
Height (cm)	127.1 ± 11	121.2 ± 13	0.20
Height SDS	−2.66 ± 0.8	−3.51 ± 1.0	0.08
BMI	17.6 ± 3.9	19.5 ± 4.0	0.17
BMI (SDS)	−0.3 ± 1.4	0.34 ± 1.1	0.12
Baseline IGF-1 SDS	−1.6 ± 1.0	−1.4 ± 1.2	0.81
Baseline IGFBP-3 (SDS)	0.0 ± 0.7	−0.4 ± 1.1	0.41
Pre-treatment height velocity(cm/year)	4.05 ± 0.6	4.07 ± 1.0	0.95

*BMI*: body mass index,* SDS*: standard-deviation score,* IGF-1*: insulin-like growth factor 1,* IGFBP-3*: insulin-like growth-factor-binding protein 3

Data are presented as mean ± standard deviation (SD). Height SDS, BMI SDS, and height velocity SDS were calculated using national Turkish reference data. IGF-1 SDS and IGFBP-3 SDS were calculated according to age- and sex-specific laboratory reference standards. *P *values represent comparisons between groups.

**Fig. 1:**
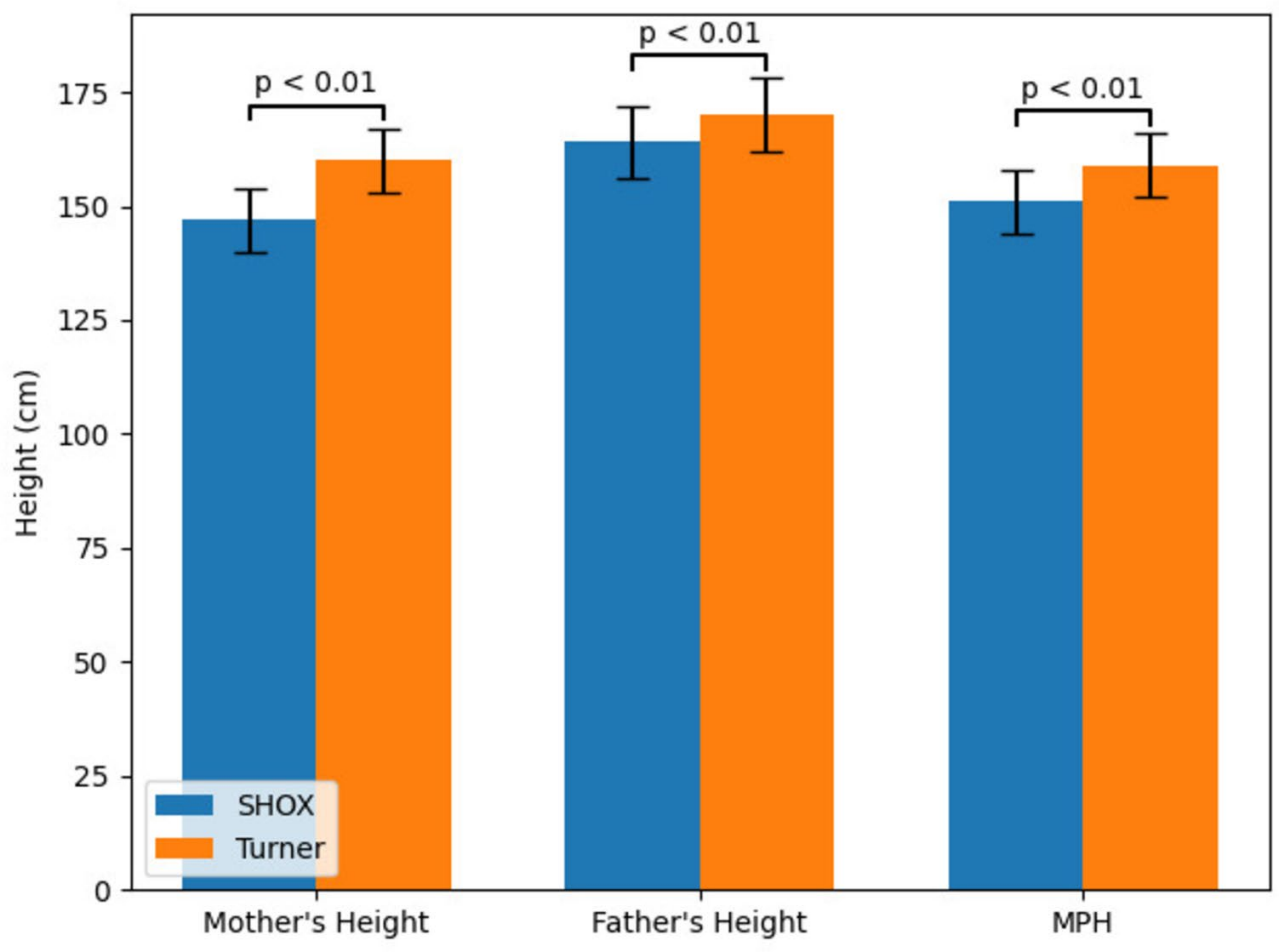
Familial stature characteristics in patients with *SHOX* deficiency and Turner syndrome (TS). Maternal height, paternal height, and calculated mid-parental height (MPH) are shown for the *SHOX* deficiency and TS groups. Bars represent mean values in centimeters, and error bars indicate ± standard deviation (SD). Parental heights and MPH were significantly lower in the *SHOX* deficiency group compared with the TS group (all *p* < 0.01)

Parental height data revealed significantly shorter maternal height, paternal height, and calculated mid-parental height (MPH) in the *SHOX* cohort compared with the TS cohort (all *p* < 0.01; Fig. 1). The mean MPH SDS was − 0.75 ± 0.8 in the TS group and − 2.37 ± 0.8 in the *SHOX* group. Segregation analysis was attempted in all 10 SHOX families; however, complete parental testing was available in only five families, in which a carrier parent was identified. Among these families, the mean height SDS was − 3.50 ± 0.2 in carrier parents and − 1.3 ± 1.0 in non-carrier parents.

### Response to growth hormone therapy and near-final height

The mean chronological age at NFH assessment was 14.2 ± 1.0 years in the *SHOX* group and 16.2 ± 1.7 years in the TS group. Both groups received comparable mean GH doses (0.045 mg/kg/day; p = 0.78). Treatment duration, however, was substantially more extended in the TS group (4.38 ± 2.3 years) than in the *SHOX* group (2.00 ± 0.4 years; *p* < 0.01). Pubertal onset also occurred later in TS patients (13.7 ± 1.5 vs 11.6 ± 1.3 years; *p* < 0.01; Table 2). All patients in the TS group required estrogen replacement therapy to induce puberty. The mean age at estrogen initiation was 13.4 ± 1.5 years.

**Table 2:** Comparative GH response and growth outcomes at near-final height in patients with *SHOX* deficiency and Turner syndrome

Variable	SHOX	Turner	P
GH dose (mg/kg/day)	0.045 ± 0.008	0.045 ± 0.009	0.78
GH-treatment duration (years)	2.00 ± 0.4	4.38 ± 2.3	< 0.01
Age at pubertal onset (years)	11.6 ± 1.3	13.7 ± 1.5	< 0.01
1st-year height velocity (cm/year)	8.55 ± 1.4	6.72 ± 1.9	0.01
2nd-year height velocity (cm/year)	6.63 ± 2.2	5.79 ± 1.5	0.20
Bone age (years)	14.1 ± 0.5	14.4 ± 0.6	0.15
NFH (cm)	150.1 ± 4.4	148.6 ± 6.1	0.39
NFH (cm)—Baseline height (cm)	23 ± 13,1	27.4 ± 14.4	0.39
NFH (SDS)	−2.46 ± 1.2	−2.47 ± 1.0	0.75
NFH (SDS)—Baseline height (SDS)	0.20 ± 0.2	1.04 ± 0.1	0.04
Height gain (cm)^b^	28.10 ± 2.0	26.04 ± 1.0	0.51
Height gain (SDS)^c^	0.77 ± 0.3	0.93 ± 0.1	0.16

*GH*: growth hormone, *SDS*: standard-deviation score *SE*: standard error, *NFH*: Near-final height

^a^Difference between near-final-height SDS and baseline height SDS (mean ± SD).

^b^Least-squares mean ± SE from ANCOVA adjusted for baseline height (cm), age at GH initiation, and treatment duration.

^c^Least-squares mean ± SE from ANCOVA adjusted for baseline height SDS, age at GH initiation, and treatment duration.

In the *SHOX* cohort, mean bone age at GH initiation was 10.6 ± 0.7 years and increased to 14.1 ± 0.5 years at treatment discontinuation. Over a mean treatment duration of 2.00 ± 0.4 years, this corresponded to a bone age advancement of approximately 3.5 years (ΔBA/ΔCA ≈ 1.75).

In contrast, in the TS cohort, bone age advanced from 9.05 ± 2.6 years at GH initiation to 14.4 ± 0.6 years at treatment discontinuation over a mean duration of 4.38 ± 2.3 years, corresponding to a ΔBA/ΔCA ratio of approximately 1.22.

**Fig. 2:**
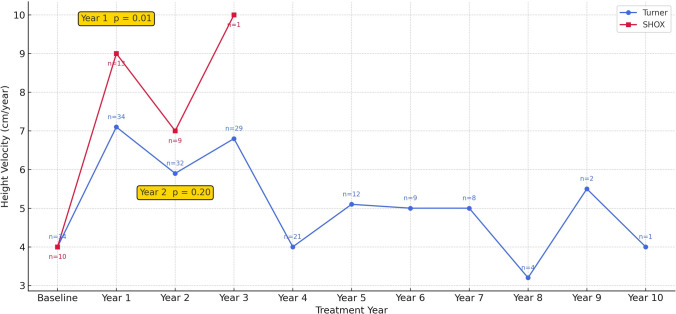
Mean Annual Height Velocity (cm/year) and Number of Cases (n). Points represent mean values. Mean height velocity (cm/year) is shown by treatment year. *SHOX* patients had a higher first-year growth velocity than TS patients (*p* = 0.01), whereas second-year velocities did not differ significantly (*p* = 0.20). *SHOX* data were available only up to year 3 due to shorter treatment duration. Numbers next to the data points indicate sample size at each time point

Annual height-velocity trends revealed a pronounced first-year acceleration in both cohorts. First-year height gain (expressed as height velocity in cm/year) was significantly higher in *SHOX* patients (8.55 ± 1.4 cm/year) than in TS patients (6.72 ± 1.9 cm/year; *p* = 0.01). Within the *SHOX* cohort, six patients entered puberty (M2) during the first year of treatment, whereas four remained prepubertal. The mean first-year growth velocity was 9.6 ± 1.2 cm/year in those who entered puberty compared with 7.0 ± 1.0 cm/year in those who remained prepubertal. However, second-year height velocities did not differ significantly between groups (*p* = 0.20). Height velocity data beyond year 2 in the *SHOX* group were limited due to shorter treatment duration, and therefore no meaningful comparison could be made for later years (Fig. 2, Table 2).

**Fig. 3:**
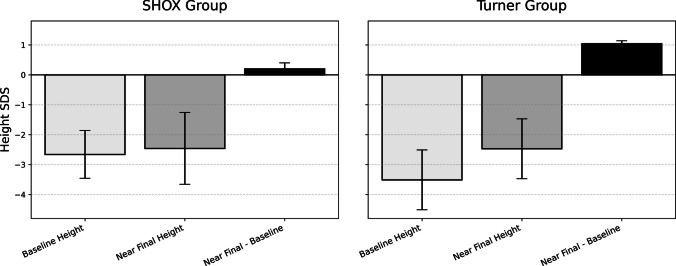
Near Final Height SDS vs Baseline Height SDS. Baseline height SDS, NFH SDS, and the change in height SDS are shown for the *SHOX* deficiency and TS groups. Bars represent mean values, and error bars indicate ± standard deviation (SD)

The mean observed total height gain from baseline to NFH was 23.0 ± 13.1 cm in the *SHOX* cohort and 27.4 ± 14.4 cm in the TS cohort (*p* = 0.39). NFH-SDS was virtually identical between groups (–2.46 ± 1.2 vs –2.47 ± 1.0; *p* = 0.75). However**,** the gain in height SDS from baseline to NFH was significantly greater in the TS cohort than in the *SHOX* cohort (*p* = 0.04; Fig. 3, Table 2)**.** Bone age at treatment discontinuation was comparable between groups (14.1 ± 0.5 vs 14.4 ± 0.6 years; *p* = 0.15).

**Fig. 4:**
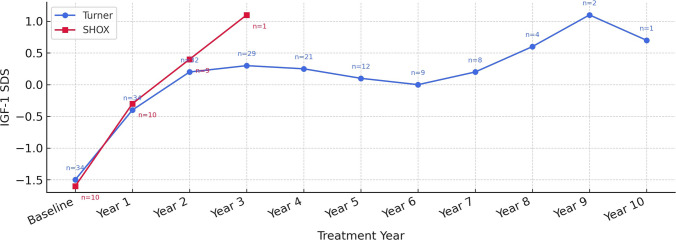
Change in IGF-1 SDS During GH Treatment. Points represent mean values. IGF-1 SDS increased in both groups following GH initiation. *SHOX* patients demonstrated a more rapid early rise, whereas TS patients showed a slower but sustained increase over time. No IGF-1 SDS values exceeded + 2. Numbers next to the data points indicate sample size

**Fig. 5:**
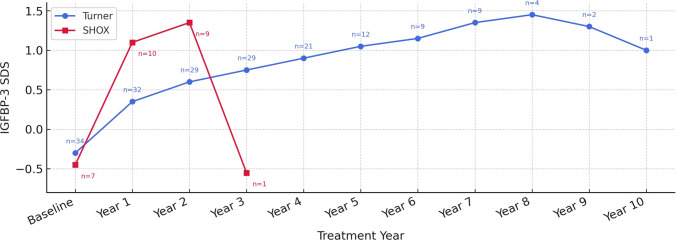
Change in IGFBP-3 SDS During GH Treatment. Points represent mean values. IGFBP-3 SDS increased in both groups during GH therapy. *SHOX* patients showed a rapid early rise, whereas TS patients demonstrated a gradual sustained increase. The apparent decline in *SHOX* after year 2 reflects the very small number of observations. Numbers next to data points indicate sample size

IGF-1 SDS rose significantly during the first treatment year in both groups. In *SHOX* patients, the increase was more pronounced during the first two years, reaching about + 1.0 SDS. In contrast, TS patients exhibited a slower, more gradual rise that eventually attained higher long-term values (Fig. 4). IGFBP-3 SDS followed a similar pattern: a marked rise through year 2 in *SHOX* patients with an apparent dip in year 3 attributable to a single outlier and a steady upward trajectory throughout treatment in TS patients (Fig. 5).

To address potential bias related to non-homogeneous baseline characteristics and treatment duration between groups, an exploratory ANCOVA model was applied. The model adjusted for baseline height SDS, age at GH initiation, and treatment duration, recognizing that treatment duration lies on the causal pathway between diagnosis and growth outcome. After adjustment, absolute height gain (cm) did not differ significantly between groups (*p* = 0.51; Table 2). Likewise, the gain in height SDS at NFH (TS 0.93 ± 0.1 vs *SHOX* deficiency 0.77 ± 0.3) showed no statistically significant difference (*p* = 0.16; Table 2). These adjusted height gain values represent estimated marginal means derived from the ANCOVA model under standardized baseline conditions and should not be interpreted as directly observed height gains.

### Safety and adverse events

No clinically meaningful adverse events occurred in either the TS or *SHOX* deficiency cohorts during GH therapy. Serum IGF-1 concentrations remained within the recommended safety range throughout treatment; specifically, IGF-1 SDS did not exceed + 2 in any patient. Consequently, no dose reductions were required, and no pharmacological toxicity or serious treatment-related complications were recorded.

## Discussion

TS and *SHOX* deficiency represent two genetically mediated causes of short stature that share a common aetiological pathway [7, 11]. In both conditions, haploinsufficiency of the *SHOX* gene impairs chondrocyte differentiation and proliferation in the growth plate, thereby restricting linear growth [13, 14]. Phenotypic expression, however, varies with the nature of the genetic defect, its mode of inheritance, and concomitant genetic or molecular modifiers [12, 17]. *SHOX* deficiency often follows a pseudoautosomal-dominant pattern and spans a broad clinical spectrum, whereas TS is a chromosomal disorder characterized by more complex systemic features [2, 17]. Comparing long-term growth outcomes after GH therapy in these two disorders may provide insight into how differences in genetic background, pubertal timing, and treatment duration shape observed growth trajectories in clinical practice. Despite their genetic differences, GH is widely employed in both conditions, and multiple determinants, including treatment age, dose, the onset of puberty, and variant type, influence the therapeutic outcome [11, 14, 18–21]. This study should therefore be interpreted as a real-world comparison of growth trajectories shaped by clinical and biological timing rather than a direct assessment of intrinsic GH responsiveness between conditions. In this context, a novel aspect of the present study is the parallel evaluation of growth outcomes in relation to differing patterns of pubertal progression: medically induced puberty in TS versus spontaneous pubertal progression in *SHOX* deficiency, both of which influence the available therapeutic window for growth.

In our cohort, maternal and paternal heights and MPH were significantly lower in the *SHOX* group than in the TS group. This likely reflects the pseudoautosomal-dominant inheritance of many *SHOX* variants, resulting in a familial pattern of short stature. Indeed, segregation analysis identified a carrier parent in five families. Similar findings have been reported by Benabbad et al., who showed that target height is often reduced in *SHOX* deficiency owing to both the mutation itself and familial factors [12]. Iughetti et al. documented a mean target-height SDS of –1.38 in *SHOX* patients [13], and Bruzzi et al. highlighted the decisive role of genotype in shaping phenotype [14].

Given the limited sample size and phenotypic heterogeneity of the *SHOX* cohort, these findings should be interpreted as descriptive, and the study is underpowered to draw strong conclusions regarding equivalence between conditions. Therefore, comparisons between groups should be interpreted cautiously, and the results should primarily be viewed as reflecting real-world growth trajectories rather than definitive differences in treatment responsiveness.

GH therapy initiated at a younger age theoretically maximizes height gain because growth plates are more responsive during the prepubertal period. Several studies on TS and *SHOX* deficiency have demonstrated an association between earlier GH initiation and higher adult height [12, 19, 22, 23]. In our study, the mean age at GH initiation was similar between groups (10.7 ± 1.9 years in *SHOX* vs 10.4 ± 2.7 years in TS) and was relatively late in both cohorts, likely reflecting delayed diagnosis in routine clinical practice. Although observed NFH in centimeters did not differ significantly between groups (*p* = 0.39), the greater unadjusted height-SDS gain in TS (*p* = 0.04) may partly reflect longer treatment duration and later pubertal onset.

First-year growth velocity was higher in *SHOX* patients (8.55 ± 1.4 cm/year) than in TS (6.72 ± 1.9 cm/year), consistent with the 8.7 ± 0.3 cm/year reported in the randomized trial by Blum et al. [21]. Long-term follow-up from the same group also demonstrated a pronounced first-year growth spurt followed by the expected decline in both conditions [19]. Similarly, second-year velocities in our cohort declined to 6.63 ± 2.2 cm/year in *SHOX* and 5.79 ± 1.5 cm/year in TS, with no significant inter-group difference. The greater first-year velocity observed in *SHOX* deficiency likely reflects the influence of spontaneous pubertal onset rather than intrinsically greater GH responsiveness. Early estrogen exposure accelerates bone maturation and epiphyseal fusion, thereby narrowing the therapeutic window for GH therapy. This mechanism may explain why the initially rapid growth velocity in *SHOX* deficiency does not translate into a meaningful cumulative height-SDS gain by NFH, particularly in patients diagnosed late and entering puberty spontaneously.

Earlier pubertal onset in *SHOX* patients (11.6 ± 1.3 vs 13.7 ± 1.5 years; *p* < 0.01) may partly explain their greater first-year velocity. Approaching puberty, GH responsiveness increases transiently under the influence of estrogen. In selected *SHOX* patients with early pubertal onset, combined therapy with GH and gonadotropin-releasing hormone (GnRH) analogues has been proposed as a potential strategy to prolong the growth period and improve adult height outcomes, although supporting evidence remains limited [11]. However, none of the patients in our cohort received such treatment.

IGF-1 and IGFBP-3 SDS rose in both groups during GH therapy. The rise in IGF-1 was earlier and steeper in *SHOX* patients. In contrast, TS patients showed a slower, progressive increase to higher long-term levels, a pattern likely related to their later puberty. Similar associations between IGF-1 dynamics and growth velocity have been reported previously [2]. In Blum’s study, IGF-1 SDS exceeded + 2 in 47% of *SHOX* and 42% of TS patients [19], whereas no IGF-1 SDS values above + 2 were observed in our cohort. Although higher IGF-1 target ranges might theoretically have resulted in greater growth responses in some patients, GH dose adjustments were primarily guided by clinical growth response and safety considerations rather than strict IGF-1–targeted titration, reflecting routine clinical practice in a real-world setting.

Literature reports adult-height gains of ~ 1.0–2.0 SDS in TS with GH therapy [7, 19, 20, 22, 24–26], consistent with the 1.04 SDS gain observed here. Our mean GH dose of 0.045 mg/kg/day corresponds to doses used in previous studies [20, 24]. The relatively late initiation of estrogen therapy in our TS cohort does not reflect current guideline recommendations, which generally advise pubertal induction around 11–12 years of age [8, 25]. Although the age at estrogen initiation varied between patients, a standardized dose-escalation protocol was generally applied throughout the TS cohort. Given the known impact of estrogen exposure on epiphyseal maturation, variability in the timing of pubertal induction may have influenced individual growth outcomes and should be considered when interpreting final height responses.

Reported height gains in *SHOX* deficiency typically range from 0.6–1.3 SDS [11, 14, 19, 20], yet the gain observed in our cohort was only 0.20 SDS. The shorter treatment duration (2.00 ± 0.4 years) and older age at GH initiation (10.7 ± 1.9 years) likely curtailed growth. Bruzzi et al. achieved a height gain of 0.80 SDS with a mean start age of 8.0 years, 5.9 years of therapy, and a dose of 0.033 mg/kg/day [14], whereas Benabbad et al. reported a gain of 1.19 SDS with a start age of 9.2 years, approximately six years of treatment, and a dose of 0.047 mg/kg/day [12]. Dantas et al. documented height gains of approximately 0.6 SDS under similar dosing, with some patients also receiving GnRH analogs [11].

In our cohort, mean bone age advanced from 10.6 ± 0.7 years at GH initiation to 14.1 ± 0.5 years at treatment discontinuation over a mean duration of 2.0 years, corresponding to a ΔBA/ΔCA ratio of approximately 1.75. This accelerated skeletal maturation is consistent with spontaneous pubertal progression and likely contributed to earlier epiphyseal fusion, thereby limiting cumulative height gain despite an initially robust growth velocity response. These findings suggest that the relatively short GH exposure in *SHOX* deficiency may reflect spontaneous pubertal progression and accelerated skeletal maturation rather than reduced intrinsic GH responsiveness or premature treatment discontinuation.

In contrast, skeletal maturation in the TS cohort progressed more gradually (ΔBA/ΔCA ≈ 1.22), consistent with delayed and medically induced pubertal development, and likely contributed to the longer duration of GH exposure observed in TS.

Exploratory ANCOVA analyses adjusting for baseline age, baseline height SDS, and treatment duration did not demonstrate a statistically significant difference between groups in absolute or SDS height gain, consistent with findings from previous multicentre studies by Blum et al. [19–21]. In those analyses, mean height gains were comparable between *SHOX* deficiency and TS (1.1 ± 0.7 vs 1.2 ± 0.8 SDS; p = 0.70) [20] and remained similar in long-term follow-up (1.34 ± 0.18 vs 1.32 ± 0.22 SDS) [19]. The adjusted analyses in our study were intended primarily as an exploratory attempt to describe height SDS gain under more standardized treatment conditions by accounting for baseline differences in age, height SDS, and treatment duration. However, these analyses should not be interpreted as evidence of intrinsic differences in biological responsiveness to GH therapy between the two conditions. Moreover, treatment duration is not an independent confounder but may lie on the causal pathway between diagnosis and growth outcome; therefore, these analyses should be interpreted cautiously. Unlike earlier multicentre trials initiated at younger ages, our real-world single-centre cohort illustrates how delayed recognition of *SHOX* deficiency and spontaneous pubertal progression may limit growth outcomes despite GH therapy.

An additional consideration relates to the use of near-final height rather than achieved adult height. Although NFH represents approximately 98% of adult height, it may not fully capture residual growth potential. This limitation may be particularly relevant in TS, where estrogen timing may influence epiphyseal maturation. In addition, NFH was reached at a younger chronological age in the *SHOX* cohort than in the TS cohort. Although height SDS values were calculated using age-specific reference standards, this difference in growth duration before NFH assessment may still influence comparisons between groups and should be considered when interpreting the results.

## Conclusion

The lower baseline height SDS in TS patients may also have contributed to the similar NFH-SDS observed between groups despite the greater height-SDS gain in the TS cohort. Differences in pubertal timing between *SHOX* deficiency and TS appear to substantially influence the duration of the available growth window and the observed response to GH therapy. Puberty occurred spontaneously in *SHOX* deficiency, whereas in TS it was medically induced at a later age, resulting in longer GH exposure in TS**.** Accordingly, these results should be interpreted as reflecting differences in growth trajectories observed in real-world clinical practice rather than intrinsic differences in biological responsiveness to GH therapy between the two conditions.

### Study limitations

This investigation has several limitations. NFH-SDS was calculated using chronological age at the time of assessment rather than projected adult height at age 18 years. Because NFH defined by BA ≥ 14 years represents approximately 98% of adult height, this approach may have led to a slight overestimation of final height, particularly in patients who reached NFH at a younger age. Although statistical adjustment was performed, the lack of exact matching between groups in terms of baseline auxological characteristics and treatment duration remains a limitation of the study.

First, the small number of patients with *SHOX* deficiency and the imbalance between the *SHOX* and TS groups reduced statistical power and restricted the scope of subgroup analyses. Because this was a retrospective single-center study, the sample size was determined by the number of eligible patients who had completed GH therapy and reached NFH during the study period, and a formal power calculation was not performed. Given the limited sample size and phenotypic heterogeneity of the *SHOX* cohort, this study does not aim to demonstrate equivalence between conditions, and all subgroup analyses should be interpreted as descriptive rather than inferential. Second, the relatively short duration of GH therapy and the use of NFH rather than adult height limited the assessment of long-term treatment efficacy. Genotype–phenotype correlations could not be fully explored because segregation analysis was unavailable for some *SHOX* cases, and LWD was diagnosed solely on clinical and physical examination findings.

In the TS cohort, patients were analyzed collectively without stratification by karyotypic subtype, making it challenging to discern differential GH responses across chromosomal variants. Pubertal onset in TS patients was often medically induced, which may have influenced growth outcomes and limits direct comparison with spontaneous puberty in *SHOX* deficiency. Although a standardized estrogen dose-escalation protocol was generally applied throughout the TS cohort, the age at pubertal induction varied between patients according to routine clinical practice. Given the known effects of estrogen exposure on skeletal maturation, this variability in pubertal timing may have influenced individual growth outcomes.

Detailed longitudinal pubertal staging data after initiation of GH therapy were not consistently available. Consequently, evaluation of bone age progression across specific pubertal stages and analysis of pubertal tempo could not be performed in a standardized manner. Interpretation of IGF-1 and IGFBP-3 dynamics was also limited, as these biomarkers are influenced not only by age and sex but also by pubertal maturation.

Furthermore, all TS patients required estrogen replacement therapy and spontaneous puberty was not observed in this cohort; therefore, a head-to-head comparison between *SHOX* patients with spontaneous puberty and TS patients with spontaneous puberty was not feasible. Finally, NFH was reached at a younger chronological age in the *SHOX* cohort than in the TS cohort. Although height SDS values were calculated using age-specific reference standards, this difference in growth duration before NFH assessment may still influence comparisons between groups and should therefore be considered when interpreting the results.

## Data Availability

The datasets analyzed in this study are not publicly available due to ethical and privacy restrictions; however, they are available from the corresponding author upon reasonable request.

## Declarations

### Clinical trial number

Clinical trial number: not applicable.
